# NIA FUNDED OBATALA SCIENCES: DRIVING DIVERSITY IN RESEARCH ENTREPRENEURSHIP AND ADVANCING REGENERATIVE MEDICINE

**DOI:** 10.1093/geroni/igae098.0490

**Published:** 2024-12-31

**Authors:** Trivia Frazier

**Affiliations:** Obatala Sciences, Inc, New Orleans, Louisiana, United States

## Abstract

Obatala Sciences, Inc. developed the first human-derived hydrogel for 2D and 3D cell culture which can be applied in various organ-on-a-chip models. The New Orleans-based Obatala Sciences fat-on-a-chip platform and supporting products offer researchers a new, more accurate way of modeling human fat tissue in-vitro. Obatala Sciences was spun out of the NIH/NIA funded LaCell LLC in 2017. To build on their prior SBIR and seed funding, Obatala Sciences raised $3 million in Series A investments in 2022. Obatala’s scientific and thought leadership are committed to promoting diversity to advance the study and prevention of diseases in the fields of obesity, diabetes, tissue engineering and regenerative medicine. In 2022, Obatala also received NIA funding to create an entrepreneurship in biotechnology training program in partnership with universities in Southeast Louisiana. The newly developed program is leveraging the diverse background and training of the diverse regional student population to give students greater insight into biotech and entrepreneurial career paths.

